# Altered potassium ATP channel signaling in mesenteric arteries of old high salt-fed rats

**DOI:** 10.20463/jenb.2016.06.20.2.8

**Published:** 2016-06-30

**Authors:** Melissa A. Whidden, Bilgen Basgut, Nataliya Kirichenko, Benedek Erdos, Nihal Tümer

**Affiliations:** 1 Department of Kinesiology, West Chester University, West Chester USA; 2 Department of Pharmacology, Near East University, Northern Cyprus Turkey; 3 Geriatric Research, Education and Clinical Center, Department of Veterans Affairs Medical Center, Gainesville USA; 4 Department of Pharmacology and Therapeutics, University of Florida, Gainesville USA; 5 Department of Pharmacology, University of Vermont, Burlington USA

**Keywords:** Potassium channels, Age, High salt diet, Vasodilation

## Abstract

**[Purpose]:**

Both aging and the consumption of a high salt diet are associated with clear changes in the vascular system that can lead to the development of cardiovascular disease; however the mechanisms are not clearly understood. Therefore, we examined whether aging and the consumption of excess salt alters the function of potassium ATP-dependent channel signaling in mesenteric arteries

**[Methods]:**

Young (7 months) and old (29 months) Fischer 344 x Brown Norway rats were fed a control or a high salt diet (8% NaCl) for 12 days and mesenteric arteries were utilized for vascular reactivity measurements.

**[Results]:**

Acetylcholine-induced endothelium relaxation was significantly reduced in old arteries (81 ± 4%) when compared with young arteries (92 ± 2%). Pretreatment with the potassium-ATP channel blocker glibenclamide reduced relaxation to acetylcholine in young arteries but did not alter dilation in old arteries. On a high salt diet, endothelium dilation to acetylcholine was significantly reduced in old salt arteries (60 ± 3%) when compared with old control arteries (81 ± 4%). Glibenclamide reduced acetylcholine-induced dilation in young salt arteries but had no effect on old salt arteries. Dilation to cromakalim, a potassium-ATP channel opener, was reduced in old salt arteries when compared with old control arteries.

**[Conclusion]:**

These findings demonstrate that aging impairs endothelium-dependent relaxation in mesenteric arteries. Furthermore, a high salt diet alters the function of potassium-ATP-dependent channel signaling in old isolated mesenteric arteries and affects the mediation of relaxation stimuli.

## INTRODUCTION

Independently, both aging and the consumption of a high salt diet are associated with clear changes in the vascular system that can lead to the development of cardiovascular disease^1-8^. Since cardiovascular disease is the leading cause of mortality in major countries, the mechanisms that contribute to the changes seen in the vasculature are of significant importance. Small resistance arteries are physiologically critical for the regulation of blood pressure and local blood flow, and it is well known that aging and a high salt diet equally induce structural and functional alterations in these arteries^9-13^. 

One of the major functional changes in the vasculature seen with both normal aging and the ingestion of large quantities of salt is endothelial dysfunction. Endothelial cells direct the tone of underlying vascular smooth muscle by releasing several factors that act to relax the vessel. Nitric oxide (NO), cyclooxygenase (COX) mediators, and endothelium-derived hyperpolarizing factor (EDHF) act to dilate resistance arteries. With age, reduced endothelium-dependent vasodilation has been characterized by reduced agonist-mediated vasodilation^14^. Similarly, young normotensive animals that are fed a high-salt (HS) diet display a dramatic attenuation in vascular relaxation responses^10, 11, 15, 16^. Endothelium-dependent vasorelaxation in the aorta and large proximal arteries is dependent almost entirely on NO^14^ while EDHF plays a role in agonist-induced vasorelaxation in distal resistance arteries including those in the mesenteric bed^17^.

Hyperpolarization through potassium channel opening is a fundamental mechanism for vasorelaxation of small vessels. ATP-dependent potassium channels are important targets of mediators released from the endothelium^18-20^. Potassium ATP channels are gated by ATP and composed of Kir^6^ subunits and a sulfonylurea receptor^21^. To our knowledge no study has evaluated the combine effect of age and dietary salt intake on the function of the KATP channel on endothelium vasodilation. Therefore, the purpose of this study was to examine the effects of age and a HS diet on endothelium relaxation in the rat mesenteric artery (MA). We hypothesized that both age and a HS diet would result in a reduction in endothelium relaxation of MAs. Furthermore, we believed that potassium ATP channel signaling would be altered in both the aged and HS fed rats. 

## METHODS

### Experimental Animals and Design

Young (5 months) and old (27 months) male Fischer 344 x Brown Norway (F344 x BN) rats were obtained from Harlan Laboratories (Indianapolis, IN, USA). The F344 x BN rat was chosen for these experiments because they live longer than the Wistar or the F344 strain in the absence of disease-specific anomalies^22^. Rats were housed individually and maintained on a 12:12 hour light-dark cycle (0600-1800 h). Experiments were conducted according to the Guiding Principles in the Care and Use of Laboratory Animals, and procedures were approved by the local Institutional Animal Care and Use Committee.

Rats were randomly assigned to one of four groups: (1) young control (standard rat diet; 15% fat, 3.1 kcal/g; diet-2018; Harlan Teklad, Madison, WI, USA; n=8); (2) young salt (8% NaCl; 15% fat; 3.0 kcal/g; TD-92012; Harlan Teklad, Madison, WI, USA; n=8); (3) old control (standard rat diet; n=8); and (4) old salt (8% NaCl diet; n=8). Animals were fed the standard and HS diet for 12 days and then over-anesthetized with pentobarbital (120 mg/kg ip). 

### Determination of Vascular Reactivity

The mesenteric artery bed was removed immediately and placed in cold modified Krebs-Ringer bicarbonate solution (that contained (in mM) 118 NaCl, 4.7 KCl, 25 NaHCO_3_, 1.2 KH_2_PO_4_, 1.2 MgSO_4_, 2.5 CaCl_2_, and 5.5 glucose). With the aid of a dissecting microscope, second-order MAs were carefully harvested. A section of the MA (~2 mm in length) was transferred to a vessel chamber, mounted, and secured between two glass micropipettes with a 10^-0^ suture. The vessel chamber was transferred to an inverted light microscope stage (Axiovert 100, Zeiss, Thornwood, NY, USA) coupled to a video dimension analyzer (Ion Optix, Corporation Hyper switch). The video dimension analyzer was connected to both a video monitor (for visualization of the vessel) and to a computer for constant recording of the intraluminal diameter of the vessel. Oxygenated (20% O_2_ – 5% CO_2_ – 75% N_2_) Krebs solution, maintained at 37°C, was continuously circulated through the vessel bath. In addition, the lumen of the vessel was filled with Krebs solution through the micropipettes. The outflow cannula was clamped off while the inflow cannula was connected to an elevated reservoir to maintain a constant intraluminal pressure of 80 mmHg. Drugs were added into the bath solution and only one concentration-response experiment was performed per arterial segment.

In order to facilitate the analysis of dilatory responses, appropriate amounts of phenylephrine (PE) were added to constrict the arteries to about 60% of their initial diameter. This provided a wide range for analysis of diameter changes during relaxation and provided a similar level of initial vascular constriction. There were no significant differences in the responses of young and old or HS fed rat arteries to PE and we added the same amount of PE to all arteries (10^-6^ M). Experimental protocols were not initiated until the vessel diameter was stable over a 15-minute period.

We tested endothelium-dependent relaxation by performing concentration-response experiments with acetylcholine (ACh; 10^-8^ M – 10^-5^ M). Typically, MAs were exposed to each dose of ACh for at least 6 minutes and maximal responses were determined. Function of the KATP channels were examined with 10 µM of glibenclamide (a selective KATP channel inhibitor) and cromakalim (10^-8^ M to 10^-4^ M), a KATP channel opener. The addition of glibenclamide to the arterial bath 10 minutes prior to ACh did not alter passive maximum internal diameters of any MAs in our groups.

The vessel diameter changes are presented as percentages (%) of dilation of the preconstricted vessels, calculated as follows: % of vasodilation = [(D_agonist_ – D_base_)/ (D_max_-D_base_)] x100, where D_max_ is the maximum diameter of the vessel during equilibration, D_base_ is the vessel diameter at steady state contraction induced by PE before the drug induced relaxation, and D_agonist_ is the diameter of the vessel after drug stimulation. With this method, the maximum dilation is represented as 100% and baseline diameter is 0%^23^.

### Chemicals

ACh, PE, and glibenclamide were obtained from Sigma (St. Louis, MO, USA), whereas cromakalim was obtained from Tocris Cookson (Bristol, UK). All drugs were dissolved in Krebs solution except for cromakalim, which was dissolved in 10% DMSO and Krebs solution. The same concentration of DMSO alone had no effect on vessel diameter.

### Evaluation of K+ Channel Expression by Western Blot

Mesenteric arteries were homogenized and assayed to quantitatively determine protein levels of KATP channel subunits. Tissue samples were homogenized in 50 mM Tris (pH 7.0) with leupeptin and protein content was assessed by the DC protein assay (Bio-Rad, Hercules, CA, USA). An equal amount of protein for each sample was separated by polyacrylamide gel electrophoresis via 12.5% gradient polyacrylamide gels containing 0.1% sodium dodecyl sulfate for ~1 h at 100 mA. After electrophoresis, the proteins were transferred to nitrocellulose membranes at 90 V for 1:30. To control for protein loading and transfer differences, membranes were stained with Ponceau S and analyzed. The membranes were washed and subsequently blocked with 5% skimmed milk in Tris-buffered saline containing 0.1% Tween 20 for 1 hr at room temperature and subsequently incubated overnight at 4°C with a primary antibody (anti-Kir6.1 or anti-Kir6.2; Santa Cruz Biotechnology Inc, Dallas, TX, USA 1:500 dilution). This step was followed by incubation at room temperature with a secondary antibody (Donkey anti-goat IgG-HRP; Santa Cruz Biotechnology Inc) for 1 hr. All bound antibodies were detected by chemifluorescence (ECL Plus Western Blotting Detection System; GE Healthcare), scanned (Storm 860 Phosphorimager Scanner; GE Healthcare) and analyzed using Image Quant software.

### Statistical Analysis

All data are expressed as means ± SE. The concentration-response curves were evaluated at each concentration for differences between young and old and control and HS diet using analysis of variance (ANOVA), followed by Tukey’s post hoc test. Significance was established at P < 0.05.

## RESULTS

Mean body weight was significantly elevated in the old rats. Prior to sacrifice, old control rats weighed 581 ± 31 g while young control rats weighed 434 ± 13 g (P<0.01). The HS diet did not alter mean body weight in either age group as young salt rats weighed 401 ± 23 g and old salt rats weighed 576 ± 14 g (Table 1). The passive maximum internal diameters at 80 mm Hg of intravascular pressure were 362 ± 10 μm, 334 ± 8 μm, 331 ± 9 μm, and 373 ± 11 μm in young control, young salt, old control, and old salt rats respectively (Table 1).

PE contracted the cannulated MAs in a concentration-dependent manner (data not shown). Pre-constriction with PE was similar in all four groups. Young MAs constricted to 60 ± 4% and 59 ± 3% of the initial diameter in the control and salt groups, respectively, while old control MAs constricted to 55 ± 4% and old salt MAs to 54 ± 5% (Table 1).

**Table 1.** Animal and vessel characteristics

	Young Control (n=8)	Young Salt (n=8)	Old Control (n=8)	Old Salt (n=8)
Age (months)	5	5	27	27
Weight (g)	434 ± 13	401 ± 23	581 ± 31*#	576 ± 14*#
Maximal vessel diameter (μm)	362 ± 10	334 ± 8	331 ± 9	373 ± 11
Constriction to PE (%)	60 ± 4	59 ± 3	55 ± 4	54 ± 5

Data are means ± SE

*Significantly increased versus Young Control (P<0.01)

#Significantly increased versus Young Salt (P<0.01)

**Figure 1.**
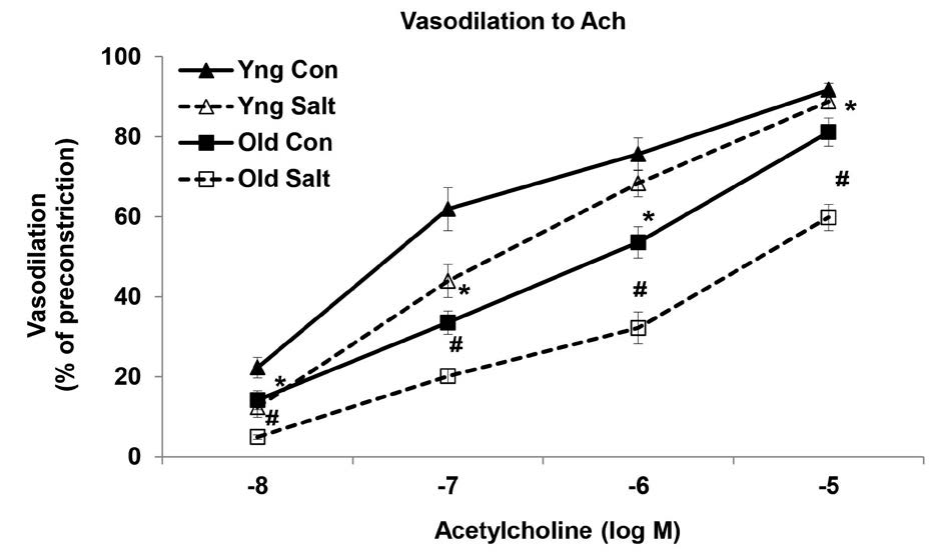
Cumulative dose-response curves to acetylcholine (ACh) in mesenteric arteries of control and high salt fed rats (n = 8/group). ^*^Significantly decreased versus Young Control (*P*<0.05). ^#^Significantly decreased versus Old Control (*P*<0.05).

ACh-induced vasorelaxation was significantly reduced in old MAs when compared with young arteries (Figure 1). Relaxation to ACh at every concentration examined (-8 M to -5 M) was significantly reduced in the old control MAs when compared to the young control arteries (P<0.05). Relaxation to ACh (10^-5^ M) was 92 ± 2% in young control MAs and only 81 ± 4% in the old control arteries (P<0.05). With regards to the HS diet, young salt animals saw a reduction in Ach-induced vasorelaxation, however it was not statistically significant. The HS diet affected the old arteries as endothelium-dependent relaxation to ACh was significantly reduced at every ACh concentration in the old salt MAs when compared with the old control arteries (P<0.05) (Figure 1). Relaxation to ACh (10^-5^ M) was 60 ± 3% in old salt MAs versus 81 ± 4% in the old control arteries (P<0.001).

**Figure 2.**
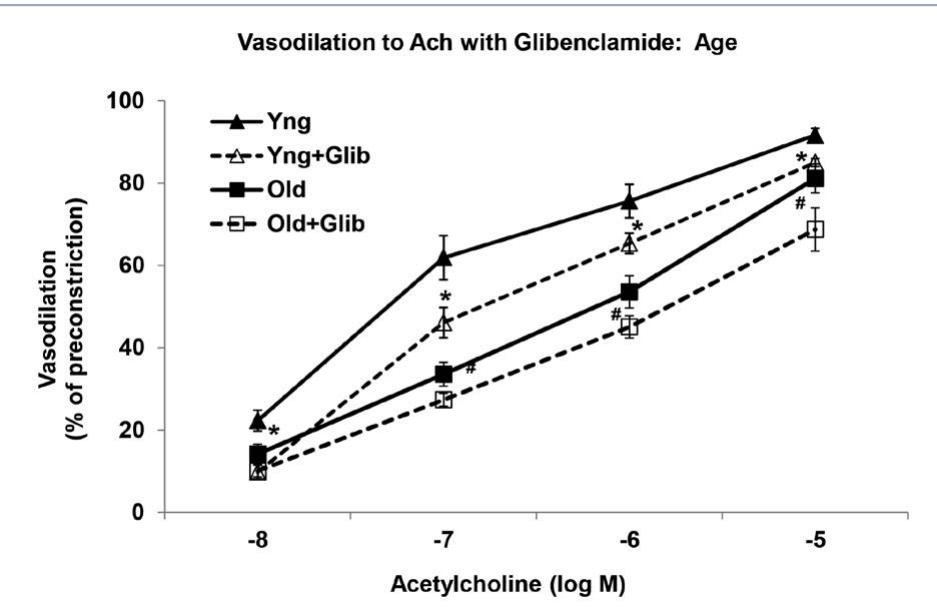
Cumulative dose-response curves to acetylcholine (ACh) with glibenclamide in phenylephrine-preconstricted mesenteric arteries of young and old rats (n = 8/group). ^*^Significantly decreased versus Young (P<0.05). ^#^Significantly decreased versus Young+Glibenclamide (P<0.05).

In young arteries, blockade of KATP channels with glibenclamide (Glib), a selective KATP channel inhibitor, significantly reduced the relaxation to ACh (-8 M to -5 M) (Figure 2). Maximum relaxations to ACh were 92 ± 2% and 85 ± 1% in the young control arteries with and without glibenclamide, respectively. In contrast, in old arteries, glibenclamide pretreatment did not have any effect on the already diminished ACh-induced vascular responses (Figure 2). Relaxations at -5 M Ach were 81 ± 3% in the old MAs and 69 ± 5% in the old MAs pretreated with glibenclamide. 

**Figure 3.**
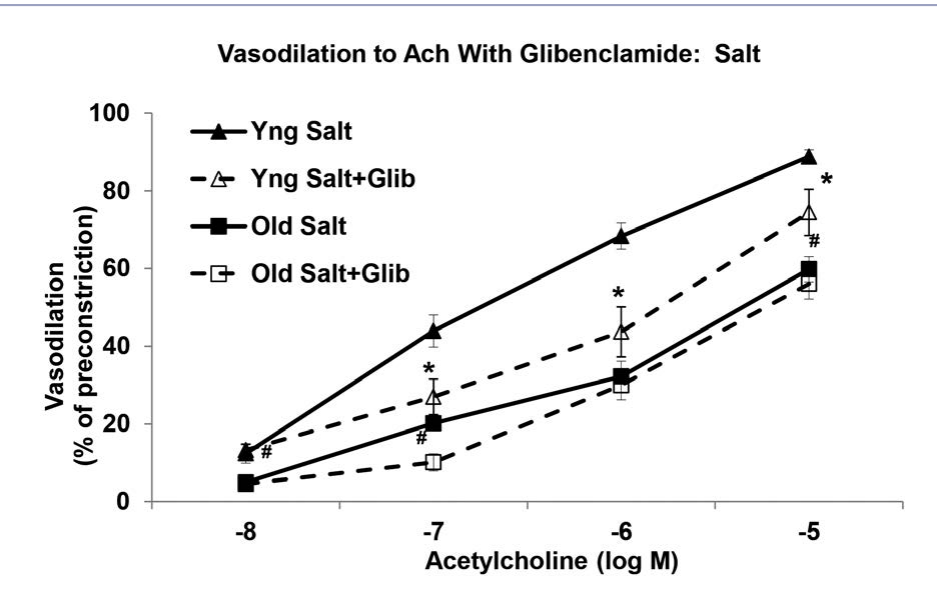
Cumulative dose-response curves to acetylcholine (ACh) with glibenclamide in phenylephrine-preconstricted mesenteric arteries of young and old rats fed a high salt diet (n = 8/group). ^*^Significantly decreased versus Young Salt (P<0.05). ^#^Significantly decreased versus Young Salt+Glibenclamide (P<0.05).

The results were similar with the HS diet. Potassium ATP channel inhibition via glibenclamide pretreatment significantly reduced the relaxation to ACh at -7 M, -6 M, and -5 M in the young salt MAs (Figure 3). Relaxations were 44 ± 4% (-7 M), 68 ± 3% (-6 M) and 89 ± 2% (-5 M) in the young salt arteries versus 27 ± 5% (-7 M), 44 ± 6% (-6 M), and 75 ± 6% (-5 M) in the young salt MAs with glibenclamide (P<0.05). On the other hand, the already diminished ACh-induced vascular response in the old salt arteries was unaffected by glibenclamide pretreatment (Figure 3). Relaxations at -5 M ACh were 60 ± 3% in the old salt MAs and 56 ± 4% in the old salt arteries treated with glibenclamide.

**Figure 4.**
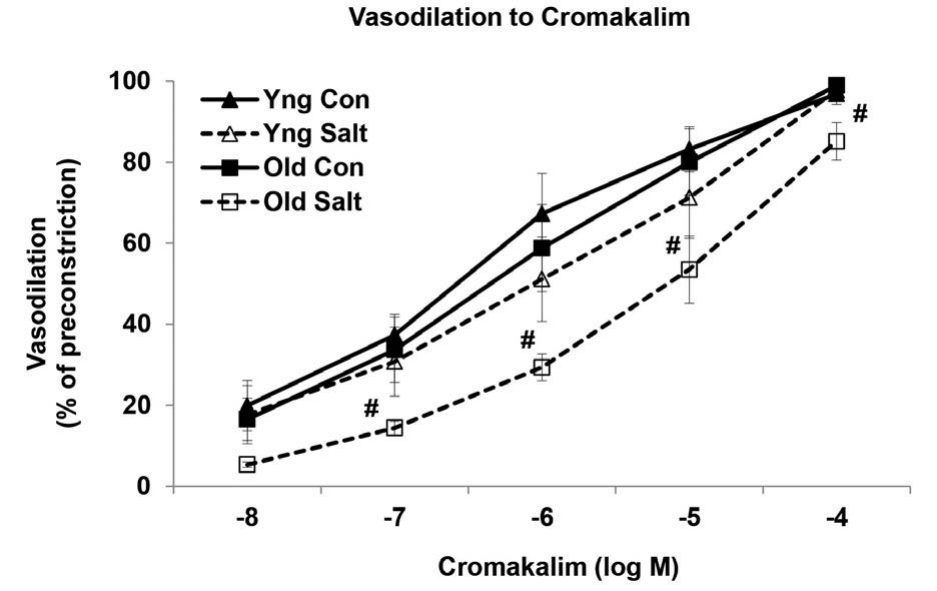
Cumulative dose-response curves to cromakalim in mesenteric arteries of control and high salt fed rats (n = 8/group). ^#^Significantly decreased versus Old Control (P<0.05).

The function of the KATP channels was examined with the specific channel opener cromakalim. Cromakalim induced dose-dependent relaxation in both the young and old MAs; and there was no difference in relaxation with age (Figure 4). However, the relaxation was markedly reduced in response to the HS diet in the old MAs (P<0.05). Maximum dilations to cromakalim (10^-4^ M) were 97 ± 3% in the young MAs versus 98 ± 1% in the young salt arteries, while dilations were 99 ± 0.7% in the old MAs when compared with 85 ± 5% in the old salt arteries (P<0.05).

**Figure 5.**
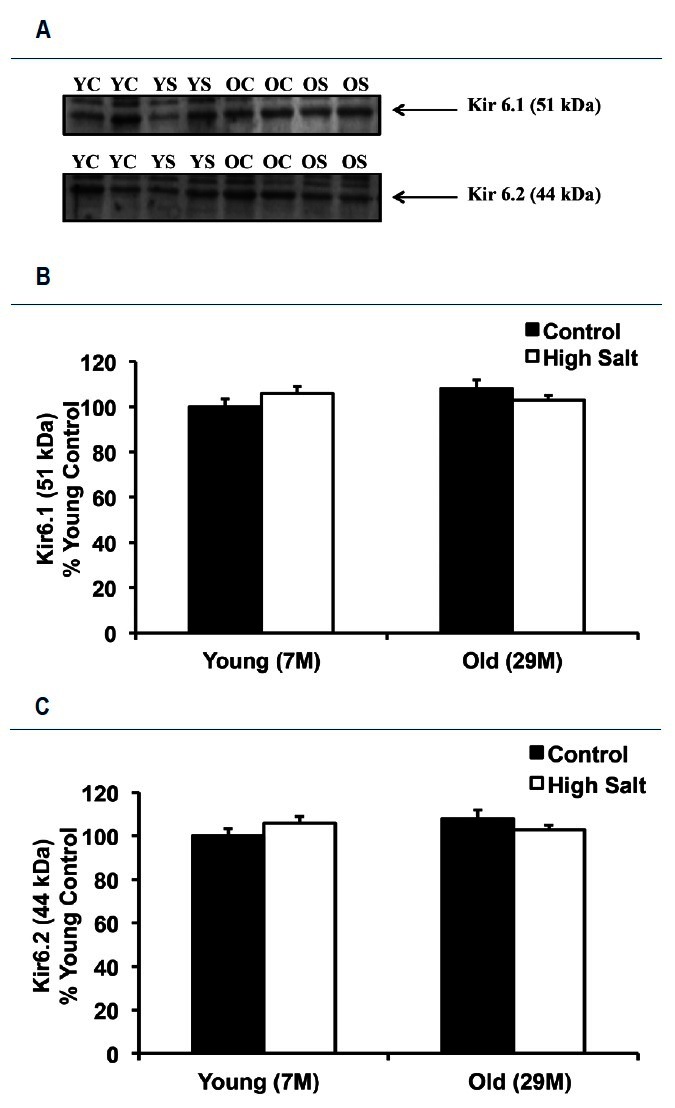
Kir 6.1 and Kir 6.2 (subunits of the KATP channel) protein abundance in mesenteric arteries. A: representative Western blots for the analysis of Kir 6.1 (51 kDa) and Kir 6.2 (44 kDa) proteins in mesentery artery samples from Young Control (YC), Young Salt (YS), Old Control (OC), and Old Salt (OS) animals. B: percent changes (vs. young control) of Kir 6.1 protein (51 kDa). C: percent changes (vs. young control) of Kir 6.2 protein (44 kDa). Values are mean percent change ± SE.

Western immunoblots with antibodies directed against the Kir6.1 and Kir6.2 subunits of the KATP channel revealed that neither age nor HS diet had any detectable effect on the expression of these proteins (Figure 5A). The Kir6.1 protein was 6 ± 3 % higher in young high salt, 8 ± 4% higher in old control, and 3 ± 2% higher in old high salt animals when compared with young control animals (Figure 5B). The Kir6.2 protein was 8 ± 2% higher in young high salt, 10 ± 3% higher in old control, and 4 ± 2 % higher in old high salt animals when compared with young control arteries (Figure 5C). 

## DISCUSSION

These experiments provide important information regarding the role of KATP channels on endothelial function in aged rats fed a high salt diet. The major findings of this study are (1) endothelium-dependent vasorelaxation is reduced in aged rats and further impaired on a high salt diet, (2) KATP channels contribute to endothelium-dependent relaxation elicited by Ach in young mesenteric arteries, however, in old arteries, the relaxation mediated by these ion channels is missing, and (3) functionally defective KATP channel signaling may contribute to the age and high salt diet-associated impairment of endothelial function. 

The aging vasculature has been extensively characterized in both large proximal arteries as well as in small resistance arteries^14, 23^. In the present study, we showed that endothelium-dependent vasorelaxation to ACh declines with age in mesenteric arteries. In vessels from aged rats, ACh-induced dilation was significantly attenuated compared to vessels from young rats, indicating an age-associated impairment of endothelial function. This finding is in agreement with Zhou et al. who showed that ACh-induced dilation was 56.25 ± 2.96% in old rats versus 94.31 ± 3.7% in young rats^23^. Similar to the study by Zhou et al., animal body weight in the present study increased with age, but we did not see an age-related increase in internal vascular diameter. The addition of the high salt diet further attenuated ACh-induced vasorelaxation but only in the aged animals. While the young mesenteric arteries were able to tolerate the excess salt (92 ± 2% control vs. 89 ± 2% high salt), the old arteries failed to relax above 60 ± 3% at 10-5 ACh. Previous studies have shown impaired ACh reactivity in middle cerebral, skeletal muscle and mesenteric arteries from rats fed a long term high salt diet^10, 11, 15, 16, 24^, however to our knowledge, this is the first study to show a combined age and salt loaded-induced attenuation of relaxation. While we did not find a statistically significant decrease in Ach-induced dilation in the young high salt mesentery arteries when compared with the young control arteries, there was still a decrease in dilatory function. A lack of significance may have been due to our small sample size. The mechanisms accounting for both the age-induced and salt loaded endothelial dysfunction are not clear but may include an increase in oxidative stress, reduced endothelial NO synthase activity and NO production^14, 25-28^. Both age and high salt diet independently increase oxidative stress in the mesenteric bed^24, 29^. However, endothelium vasodilation in mesenteric arteries is not entirely dependent on NO production^23^.

KATP channels play an important role in agonist-induced vasorelaxation in small resistance arteries. Opening of potassium channels on vascular smooth muscle cells with resultant hyperpolarization plays a central role in distal resistance arteries. ACh has been shown to activate these ion channels in several circulatory beds. In the present study, we used glibenclamide to block KATP channel opening and in young (control and high salt) arteries, ACh-induced relaxation was significantly reduced. This finding provides evidence that KATP channels contribute to ACh-induced dilation in young rat mesenteric arteries. However, the already reduced ACh-induced relaxations in old (control and high salt) arteries were not further affected by the application of glibenclamide. Thus the present data demonstrates that attenuated ACh-induced dilation in old animals appears to be due to impaired KATP channel signaling and/or activation. 

It is not clear at this time what kinds of mechanisms are responsible for the aging-induced KATP channel impairment. Theoretically, both the reduced release of dilator stimuli and the dysfunction of vascular smooth muscle K+ channels activated by these mediators can affect vasorelaxation. Here we demonstrate that relaxation to cromakalim is only significantly reduced in the mesenteric arteries of old rats fed a high salt diet compared with old animals fed a control diet. This finding clearly indicates that KATP channel mediated dilation was impaired in the old high salt fed animals even when the ion channel was stimulated by a specific channel opener. Obiefuna et al. demonstrated that KATP channels are deactivated in smooth muscle cells of the thoracic aorta in salt-induced hypertension^30^. To further examine potassium ATP channel function with age and excess salt consumption, we analyzed the expression of and, as a result, the density of KATP channels in the mesenteric arteries, leading to reduced total K+ efflux when widespread channel activation occurs. However, using immunoblot analysis, we found that levels of the pore-forming subunits of the KATP channels are not detectably affected by either age or a high salt diet. Thus a high salt diet alters the function of KATP channel signaling in the regulation of stimulated vascular control mechanisms in aged animals.

Further investigation is needed to reveal how the changes in old high salt fed rats affect the function of these vascular smooth muscle cell K+ channels. One possibility is that age in combination with a high salt diet increases the production of reactive oxygen species (ROS) in the vascular tissue, which influences the structure and activity of these ion channels. The following evidence supports this hypothesis. First, superoxide production has been shown to be elevated in mesenteric arteries of both old and high salt fed rats. Second, K+ channel mediated relaxations are impaired in pathophysiological conditions when excessive ROS production occurs, for example, during ischemia-reperfusion^31, 32^, brain injury^33, 34^, or hyperglycemia^35, 36^. Thus the impairment of potassium ATP channel mediated vascular response in old high salt fed mesenteric arteries could also be the consequence of elevated ROS production.

In conclusion, results of the present study indicate that excess salt consumption during normal aging significantly impairs the function of KATP channel signaling in rat mesenteric arteries, and the altered signaling of these channels influences the mediation of endothelium-dependent relaxation. The altered vascular regulation may be responsible for elevated blood pressure and increased risk for cardiovascular events in the elderly population especially those individuals who consume excess salt on a daily basis.
